# Dietary Habits and Eating Practices and Their Association with Overweight and Obesity in Rural and Urban Black South African Adolescents

**DOI:** 10.3390/nu10020145

**Published:** 2018-01-29

**Authors:** Modiehi Heather Sedibe, Pedro T. Pisa, Alison B. Feeley, Titilola M. Pedro, Kathleen Kahn, Shane A. Norris

**Affiliations:** 1 Department of Human Nutrition, School of Health Care Sciences, Faculty of Health Sciences, University of Pretoria, Pretoria 0001, South Africa; 2 MRC/Wits Developmental Pathways for Health Research Unit, School of Medicine, University of the Witwatersrand, Johannesburg 2198, South Africa; pppedropissa@gmail.com (P.T.P.); alison.feeley@gmail.com (A.B.F.); titilolapedro@gmail.com (T.M.P.); shane.norris@wits.ac.za (S.A.N.); 3 UNICEF Equity House, Brooklyn, Pretoria 0181, South Africa; 4 Epidemiology and Global Health Unit, Department of Public Health and Clinical Medicine, Umeå University, 901 85 Umeå, Sweden; kathleen.kahn@wits.ac.za; 5 INDEPTH Network, East Legon, Accra 23321, Ghana; 6 MRC/Wits Rural Public Health and Health Transitions Research Unit (Agincourt), School of Public Health, Faculty of Health Sciences, University of Witwatersrand, Johannesburg 2198, South Africa

**Keywords:** dietary habits and practices, adolescents, overweight, obesity, South Africa

## Abstract

The aim of this study was to investigate differences/similarities in dietary habits and eating practices between younger and older, rural and urban South African adolescents in specific environments (home, community and school) and their associations with overweight and obesity. Dietary habits, eating practices, and anthropometric measurements were performed on rural (*n* = 392, mean age = 13 years) and urban (*n* = 3098, mean age = 14 years) adolescents. Logistic regression analysis was used to examine the associations between dietary habits and eating practices, with overweight and obesity risk. Differences in dietary habits and eating practices by gender and by site within the three environments were identified. After adjusting for gender, site, dietary habits, and eating practices within the home, community and school environment, eating the main meal with family some days (OR = 1.78, 95% CI = 1.114–2.835; *p* ≤ 0.02), eating the main meal with family almost every day (OR = 1.61, 95% CI = 1.106–2.343; *p* ≤ 0.01), and irregular frequency of consuming breakfast on weekdays (OR = 1.38, 95% CI = 1.007–1.896; *p* ≤ 0.05) were all associated with increased risk of overweight and obesity. For “Year 15” adolescents, irregular frequency of consuming breakfast on weekends within the home environment (OR = 1.53, 95% CI = 1.099–2.129, *p* ≤ 0.01), was associated with increased risk of overweight and obesity. For both early- and mid-adolescents, being male (OR = 0.401, 95% CI = 0.299–0.537; *p* ≤ 0.00; OR = 0.29, 95% CI = 0.218–0.397; *p* ≤ 0.00) was associated with reduced risk of overweight and obesity, while residing in a rural setting (OR = 0.55, 95% CI = 0.324–0.924; *p* ≤ 0.02) was associated with reduced risk of overweight and obesity only among early-adolescents. Only dietary habits and eating practices within the home environment were associated with increased risk of overweight and obesity.

## 1. Introduction

The prevalence of obesity in both high-income and low- and middle-income country populations is increasing [1,2]. Among adolescents, the occurrence of overweight and obese individuals has drastically increased over the past 20 years [3,4], a phenomenon that has been attributed to modifiable lifestyle factors linked to diet, physical activity, and sedentary behaviour [5].

Being overweight or obese during adolescence has been shown to increase one’s risk of developing non-communicable diseases (NCDs) in adulthood [6]. Research also suggests that dietary habits and practices developed in childhood are usually maintained into adulthood, and this predisposes individuals to obesity and increased metabolic disease risk [7,8]. The link between a higher body mass index (BMI) and a lower self-esteem has been shown, but the opposite is not always true. The degree of being overweight in girls has been shown to be inversely correlated to their level of physical self-esteem [9,10,11]. Among urban South African adolescents poor eating habits within the home, community and school environment, with poor dietary patterns had already been established in early adolescence [12].

Differences and similarities in dietary habits and practices, and their association with overweight and obesity risk in urban and rural South African adolescents are not known. In addition, little is known about the differences in dietary habits and practices at different adolescent stages (early vs. mid adolescence). Therefore, the aim was to compare the different dietary habits and eating practices between rural and urban adolescents in specific environments (community, home and school environment) and their associations with overweight and obesity.

## 2. Materials and Methods

### 2.1. Setting and Study Population

Data for the current study was collected from two sites, Agincourt (rural): Health and Demographic Surveillance System (AHDSS) and Soweto (urban): Birth to Twenty (Bt20) cohort, between the years 2008–2009.

#### 2.1.1. Rural Site: Agincourt—AHDSS

This study was conducted in rural Agincourt, a sub-district of Bushbuckridge, Mpumalanga province, northeast of South Africa, with an approximate population of 70,000. The study site lies close to the border with Mozambique, bordering the Kruger National Park conservation area. This site provides the foundation for the Rural Public Health and Health Transitions Research Unit of the Medical Research Council (MRC), and the University of the Witwatersrand, South Africa (the MRC/Wits-Agincourt Unit). Further details of the study site have been published elsewhere [13,14].

#### 2.1.2. Urban Site: Soweto—Bt20 Cohort

The Birth to Twenty (Bt20) cohort monitors children’s health and wellbeing [15] and is comprised of only singleton children (*n* = 3273) born between April and June 1990, resident for at least six months in the Soweto-Johannesburg municipality after birth, and whose parents gave consent to be enrolled in the study. The cohort is demographically representative of long-term residents of Soweto-Johannesburg, and has been followed from birth to 23 years of age [16,17]. Soweto is a township of approximately 1.2 million people in the city of Johannesburg, South Africa. Attrition over two decades has been comparatively low (30%), mostly occurring during the children’s infancy and early childhood. Descriptive details of the study site have been published elsewhere [16,17]. In the Birth to Twenty cohort, data collected at ages 13 and 15 years was used in the current study.

The sampling and recruitment for this study in rural Agincourt was done through the AHDSS. According to the statistical sample size calculation, 600 children from the AHDSS were selected to participate in the 2009 data collection, which was termed the Growth 2 Study. Cross sectional data was collected from participants who were 11–12 years of age (mean age 11.5) and those who were 14–15 years of age (mean age 14.5). Previous studies have published details of recruitment methods and study design [13,14].

Participants were divided into two age groups: the “early adolescence” age group (Year 13), which represents the onset of adolescence; and the “mid-adolescence” age group (Year 15), in order to identify/detect any differences in dietary habits and eating practices at different age groups across the different domains of influence.

Adolescence may be divided into three developmental stages based on physical, psychological and social changes: early adolescence, 10/13–14/15 years; mid adolescence, 14/15–17 years; and late adolescence, between 17 and 21 [18]. Participants from the Agincourt site and BT20 cohort who were 11/12 and 13 years of age were respectively grouped into the “early-adolescence” group and were compared. Participants from the Agincourt site and BT20 cohort who were 14/15 and 15 years of age were respectively grouped into the “mid-adolescence” group and compared.

#### 2.1.3. Ethics

Ethics clearance for both Agincourt (M090212) and Soweto (M080320) sites were obtained from Witwatersrand University Committee for Research on Human Subjects. Ethical Committee of Mpumalanga Province gave additional clearance for Agincourt site. Primary caregivers gave written informed consent for their child to participate in the research at each assessment visit, and the child provided written assent. Confidentiality was maintained by the allocation of an identification number for each participant which was used on all questionnaires.

#### 2.1.4. Dietary Habits and Eating Practices Assessment

The questionnaire used in this study was developed with the guidance of a literature review. Questions around dietary habits and practices which have been shown to be associated with poor nutritional outcomes [19,20,21,22] within three key settings (home, school and community) were formulated. The questionnaire was similar to a non-qualified food-frequency questionnaire, where the recording of the frequency of certain food items over the recall period was done. Eating practices that participants engaged in were identified, and once determined, food eaten (from a predetermined list based on focus group findings) and the frequency of the practice in the previous week was also investigated. This way the engagement in eating behaviour (and, where relevant, the foods consumed and their frequency) was investigated.

In both sites, the fieldworkers were trained on the administration of the questionnaire.

The questionnaire used was translated into local languages (including seSotho, isiZulu, and Shagaan/Tsonga), piloted, and modified in fieldwork debriefing sessions to ensure meaning equivalence of the questions. Piloting was carried out on a group of adolescents conveniently sampled to ensure understanding of questions and to test for appropriate translation to local vernacular. Reliability was assessed by using the test-retest design method, administering the questionnaire to the pilot participants (*n* = 20) twice, one week apart. To determine the retest reliability kappa-coefficients for nominal data were used, which showed very strong agreement between the first and second test responses, ranging from 0.89 to 1.00 for the different questions.

An interviewer administered the questionnaire during the participants’ visit to the data collection sites in Soweto and Agincourt. The questionnaires covered dietary habits and eating practices, and eating occurring in three environments/settings: in the home, in the community, and in the school setting. In the home setting, participants were asked how frequently they ate their main meal with their family (“never”, “some days”, “almost every day” = most days/every day); categorised as follows: irregular = “never/some days” and regular = “most/everyday”; how frequently breakfast was consumed during the previous week, coded as irregular (≤2 days/week) or regular (3–5 days/week), and on the weekend, coded as irregular (0–1 weekend day) or regular (both weekend days); how frequently snacks were consumed while watching television (TV) in the previous week, coded as irregular ≤ 3 times/week, regular ≥ 3 times/week; and the number of snacks consumed while watching TV in the previous week (0, 1, 2, 3, 4 or >5 snacks/week).

In the community setting, participants were asked how frequently they consumed fast foods (irregular ≤ 3 times/week, regular ≥ 3 times/week) and the number of fast food items (0, 1, 2, 3, 4, >5) consumed in the previous week. In the school setting, participants were asked how frequently they made tuck shop (vendors and retailers on school premises) purchases (coded as irregular ≤ 3 times/week or regular > 3 times/week), and the number of items (0, 1, 2, 3, 4, >5) purchased at the tuck shop (TS) during the previous week; and how many days during the previous week a lunch box (LB) (irregular ≤ 3/week, regular > 3/week) was used [23].

#### 2.1.5. Anthropometry

Anthropometry measurements were collected by trained fieldworkers. Weight (digital scale from Dismed, Miami, FL, USA) to the nearest 100 g, and height (stadiometer from Holtain, Crosswell, Crymych, UK) to the nearest millimetre, were measured for all participants, with subjects wearing light clothing and no shoes. A software program (AnthroPlus 2007) developed by the World Health Organization was used to calculate Z-scores for BMI for age and constitute growth reference data for children aged 5 to 19 years [24]. A Z-score of −2 is recommended as a cut-off for thinness. Cut-off scores for overweight and obesity are +1 and +2, respectively. The BMI (weight in kilograms divided by height in meters squared) (kg/m^2^) was computed and BMI-for-age Z-scores were computed to determine overweight and obesity among adolescents using WHO 2007 BMI-for-age Z-score cut-off [24]. This coincides, at 19 years of age, with adult cut-offs for BMI of 25 and 30 respectively. For the purpose of this paper the adolescents were divided into underweight (Z ≤ −2SD), normal (Z > −2 ≤ +1SD), overweight (Z > +1 ≤ +2SD), and obese (Z > +2SD). In the bivariate regression models, the adolescents were divided into two categories: normal (Z > −1.99 ≤ +1SD), overweight and obese (Z > +1SD).

#### 2.1.6. Statistical Analysis

All statistical analyses were performed using STATA statistical software package version 10.0 (StataCorp LP, College Station, TX, USA). Descriptive statistics were performed for each variable. For continuous variables, the Bartlett’s test/Student *t*-test was used to test the differences between means across gender within the same age group, within the different study sites. For categorical variables frequencies are presented and the Pearson χ^2^ test was run for differences in proportions by gender within specific age groups, in different sites. For comparing groups with less than five participants in each group, in addition to the Pearson χ^2^ test, the Fisher’s exact test was done. The results of the two did not differ (Pearson χ^2^ test vs. Fisher’s exact test), thus only results from the Pearson χ^2^ test are presented. Logistic regression analyses were conducted to test the association between dietary habits and eating practices in different environments with risk of being overweight or obese for early-adolescents (EA) and mid-adolescents (MA). Odds ratios (95% CI) were computed for four models. The crude model adjusted for gender and site, M1: (included the crude model plus home dietary habits and eating practices), M2: (included M1 plus and community dietary habits and eating practices), M3: (included M2 plus school dietary habits and eating practices) and M4 (included M3 plus school dietary habits and eating practices). R2 values are presented for each model. A *p*-value of <0.05 was considered statistically significant.

## 3. Results

Descriptive characteristics of participants at EA and MA are presented in Table 1 by gender and site [rural (Agincourt) and urban (Soweto)]. In both adolescent groups and at both sites, mean BMI was significantly higher in girls. EA urban boys and girls had a significantly higher mean BMI compared to their rural counterparts (*p* ≤ 0.00), and there were significantly more urban boys and girls in the overweight and obese BMI-for-age Z-score cut-offs (*p* = 0.00) category, and this difference was not observed in the MA group. Based on BMI-for-age Z-score cut-offs, after combining the numbers in the overweight and obese category, more females were in this category at both sites as compared to boys at both EA (rural: 17.34% vs. 9.52%; urban: 36.15% vs. 27.89%) and MA (rural 22.33% vs. 5.50%; urban: 28.5% vs. 12.82%). The urban site had more overweight and obese adolescents as compared to the rural site. In the urban site, more EA (5.39% vs. 3.73) and MA (7.44 vs. 2.11%) boys than girls, respectively, were underweight.

### 3.1. Dietary Habits and Eating Practices

#### 3.1.1. Home Environment

Significantly more girls than boys ate their main meal with the family “almost every day” in the previous week, at both EA (51.02% vs. 33.33%) and MA (48.51% vs. 28.57%) in the rural site, whereas this was not observed in the urban site (Table 2 and Table 3). The frequency of those eating their main meal with family some days was higher among rural vs. urban EA (44.33 vs. 14.07%, *p* ≤ 0.00) and MA (38.54% vs. 26.88%, *p* ≤ 0.00) participants, respectively (Table 4). Irregular breakfast consumption on weekdays was significantly higher in EA and MA girls (29.25% & 44.89%) vs. males (21.19 & 27.47%), in the urban site. Urban girls consumed more snacks while watching TV than boys at both (5 ± 5.6 vs. 4 ± 5.1; *p* ≤ 0.02) and MA (9 ± 7.3 vs. 8 ± 5.9, *p* ≤ 0.000); this was not observed in the rural site (Table 2 and Table 3). Significantly more urban than rural participants consumed snacks regularly while watching TV (49.46% vs. 18.72%) only at EA (Table 4).

#### 3.1.2. Community Environment

At MA, girls from the urban site, consumed significantly more fast food items than boys (18 ± 9.9 vs. 17 ± 8.9, *p* ≤ 0.00), respectively (Table 3). No differences between boys and girls were observed in terms of fast foods consumed at EA and MA in the rural setting.

#### 3.1.3. School Environment

Tuck shop: In the urban site, EA girls purchased significantly more tuck shop items in the previous week (13 ± 7.9 vs. 12 ± 8.0, *p* ≤ 0.00) (Table 2), which was not observed for among EA and MA adolescents from the rural setting (Table 2 and Table 3).

Lunch box (lunch brought from home): At EA, all participants (both boys and girls) had irregular lunch box usage in the previous week, at both sites. However, at MA, the frequency of lunch box usage in the previous week was more irregular among boys (MA, 92.25% vs. 81.9% *p* ≤ 0.00) in the urban site, no differences were observed in the rural setting (Table 3).

#### 3.1.4. Association with Overweight and Obesity Risk

Table 5 and Table 6 present the odds ratios for being overweight and obese with gender, site, and dietary habits and eating practices in different settings (home, community and environment) at EA and MA respectively. Results of the crude model (adjusted for gender and site) are not shown in Table 5 and Table 6. In M4, being male (OR = 0.40, 95% CI = 0.299–0.537; *p* ≤ 0.00; OR = 0.29, 95% CI = 0.218–0.397; *p* ≤ 0.00) was associated with reduced risk of being overweight and obese at EA and MA, respectively. Residing in the rural site (OR = 0.55, 95% CI = 0.324–0.924; *p* ≤ 0.02) was associated with a reduced risk of being overweight and obese only among EA participants. Eating the main meal with family some days (OR = 1.778, 95% CI = 1.114–2.835; *p* ≤ 0.02); eating the main meal with family almost every day (OR = 1.61, 95% CI = 1.106–2.343; *p* ≤ 0.01); and irregular frequency of breakfast consumption on weekdays (OR = 1.38, 95% CI = 1.007–1.896; *p* ≤ 0.05); were associated with increased risk of being overweight and obese among EA; whereas among MA participants, irregular frequency of breakfast consumption on weekends (OR = 1.53, 95% CI = 1.099–2.129, *p* ≤ 0.01) was associated with increased risk of being overweight and obese.

## 4. Discussion

This study investigated differences/similarities in dietary habits and eating practices between urban and rural adolescents, and also between younger and older adolescents, in specific environments (home, community and school environment) and their associations with overweight and obesity. At early and mid-adolescence, more girls were in the overweight and obese category than boys in both sites, and the proportion of overweight and obese adolescents was higher in the urban site.

### 4.1. Dietary Habits and Eating Practices

#### Home

Main meal with family: Site differences were observed across the two adolescence stages. For urban adolescents, more males than females ate their main meal with the family “almost every day” in the previous week. In the rural setting, more female adolescents than males ate their main meal with the family “almost every day”. These findings compare with observations made among Malaysian and USA female adolescents [25,26]. A USA-based study reported that greater involvement in food preparation was associated with increased frequency of meals taken with family [27]. Adolescents involved in food tasks were also more likely to consume excess energy or exercise less often than their peers [27], which could also explain the increased prevalence of overweight and obesity among female adolescents in the current study communities [28]. The difference between the urban and rural adolescents eating the main meal with the family can also be explained by the local rural [29] practice (which also occurs in USA communities) where adults within the same family do not always eat at the same time or in the same room with the children [27,30]. There is an inherent cultural tendency to motivate and persuade other family members to finish their food and eat more. However, this was not observed in urban areas, and this could be linked to them being more knowledgeable about the consequences of over nutrition and obesity risk.

Breakfast: In both sites and genders, more than 60% of participants in early and mid-adolescence consumed breakfast regularly during the week. These findings are in line with European and local findings [31,32,33], and such participants had the lowest proportion of obesity [34]. Site differences observed where 25% urban vs. 8.87% rural early-adolescents, consumed breakfast irregularly, are comparable, and at times higher than recent national findings of 14.4% and 9.8%, urban and rural, respectively [31]. In terms of those that consumed breakfast irregularly, current data compares with findings among 12–17 year-old Cape Town-based black South African adolescents, where 22% did not have breakfast before school [27], and in USA-based studies where at least 19% of adolescents skipped breakfast [35,36]. It is important to also highlight that the current study participants were not asked where they ate breakfast, and whether they ate food from home or outside the home for breakfast. Based on recent research conducted among females in the current urban setting, the majority bought unhealthy food items such as the “vetkoek” (fried dumplings made from wheat flour) just before school from vendors, which could account for the higher percentages of participants who skipped breakfast [37]. The majority of urban participants cited time limitations as a reason for not consuming breakfast before going to school, even though they knew its importance. They, however, reported eating fast foods and snacks sold by school vendors as breakfast options [37]. In the current rural setting, the majority of females cited unavailability of food at home as a reason for not eating breakfast [29]. In other South African studies, the majority of participants reported not being hungry in the early morning, not having enough food in the house, or other people within the household not having breakfast, as the most common reasons for skipping breakfast [31].

Snacks: Gender differences were observed in the urban site where girls consumed more snacks while watching TV than boys at both early- and mid-adolescence. In terms of site, significantly more urban than rural participants consumed snacks regularly while watching TV only at early-adolescence. Urban participants also consumed more snacks while watching TV than rural participants at both early- and mid-adolescence. Current findings are in line with previous studies, where television watching among girls was significantly associated with more snack consumption, which was also a strong predictor of increase in BMI [38]. Previous longitudinal studies among children [39] and adolescents in early- and mid-adolescence [40] have shown a significant association between the availability of snacks and the consumption thereof within the home environment. In the current study, it is likely that snacks are more available within the urban vs. rural adolescents’ household owing to socio-economic status factors.

### 4.2. Community

Fast foods: Across both sites and genders, almost all participants consumed fast foods regularly in the week prior to data collection. A gender and site difference was only observed among mid-adolescent participants, where urban girls consumed significantly more fast food items than boys, but no significant difference was observed across gender and site among rural participants in the two adolescent stages.

These findings were different from recent national surveys in South Africa [31,41] and USA findings among adolescents and young adults [42]. Based on other research, the most frequently consumed fast food items in Soweto and Agincourt were the local energy-dense kota (quarter-loaf of white bread filled with fried potato chips, processed cheese and any other processed meats and sauces), chips, and vetkoek (fried dumpling made from wheat), due to their affordability and accessibility [29,37]. Among urban females, these options were also cited as breakfast and meal replacements consumed at household level [37].

### 4.3. School

Tuck shop: Most participants in the urban and rural sites made purchases from the school tuck shop, with significantly more mid-adolescent girls than boys making tuck shop purchases. This difference was not observed among early adolescents. In a cross-sectional survey of USA adolescents from 20 schools (grades 9–12), 72% of participants did not use lunch boxes in the previous week, and 31% reported purchasing snacks from the school vending machine [43].

These findings match those from adolescents in Cape Town, where significantly more girls (74.6%) than boys (62.9%) bought food from school, and the majority (85.7%) of the purchases were unhealthy [33]. However, the high rate of tuck shop purchases in the current study exceeds findings from other local studies [31,33]. Recent findings in the current communities show that most food items purchased at tuck shops are unhealthy, but females still prefer to buy food rather than bring food to school, with purchases being influenced by cost and availability [29,37]. This suggests that if the quality of food at school tuck shops and vendors could be improved, the general diet of adolescents could be enhanced.

Lunch boxes: Irregular lunchbox use was common among mid-adolescent (>70%) and early-adolescent participants (100%) in the two sites. These figures are comparable to, and even higher than found in recent national data where 62.4% of children aged 10–14 years took lunch boxes to school sometimes or not at all [31]. In the SANHANES-1 study, the proportion of African participants aged 10–12 years of age who “did not/sometimes took” lunch boxes to school was at 66.9% vs. 62.5% at the national level [31]. More girls than boys among mid-adolescent urban participants in the current study used lunch boxes regularly in the previous week. Significantly more urban than rural participants were irregular lunch box users among mid-adolescents. This is different from current national findings where generally more rural than urban participants did not take a lunch box to school or did so only sometimes [31]. These findings are also in line with the SANHANES-1 study where there were no significant differences in lunchbox practice by gender in children aged 10–14 years [31]. Current study findings are higher than the SANHANES-1 results where more urban (40–47.6%) vs. rural (25.3%) dwellers aged 10–14 years of age, did not take a lunch box to school [31]. Previous findings among rural female adolescents in the current study showed school feeding programmes as enablers of healthy eating in local high schools [29].

### 4.4. Association with Risk of Being Overweight or Obese

Male participants in early and mid-adolescence were less likely to be overweight and obese compared to their female counterparts. In the current study, being rural and male was associated with a lowered likelihood of being overweight and obese in early- and mid-adolescence. Based on previous findings in the rural setting under investigation, the majority of adolescents walked long distances to and from school as opposed to their urban counterparts. Most females also engaged in household chores: urban females tend to engage in fewer household chores [37] than their rural adolescent counterparts resulting in increased physical activity for the rural female participants [29]. These findings were however different from USA-based studies where children living in rural settings were more likely to be overweight than their urban counterparts [44,45,46,47], but in a nationally representative study of children ages 5–17 years, the risk of being overweight and obesity among rural female participants was mostly linked to physical inactivity, television watching, and computer use [48].

Related to dietary habits and eating practices within the home: irregular breakfast consumption during the week and weekends and eating the main meal with family “some days” and “almost every day”, accounted for most of the increased risk of overweight and obesity.

The current findings are in agreement with findings among adolescents in Dubai, UAE, Brazil, and the Gulf region, where skipping breakfast was significantly associated with increased overweight and obesity among girls [49,50,51], and USA and Europe-based longitudinal studies where breakfast skipping among adolescents was associated with an increased risk of being overweight [52,53]. Among females, this could also be a reflection of a dieting technique in which the female students skipped breakfast as means of weight control [29]. This technique is most prevalent among girls as observed among female students aged 18 to 24 years in USA and local studies [54]. This is a practice that starts in early adolescence and remains prevalent throughout adulthood [55]: it has also been found in other local studies [37] and investigations in developed countries [54]. Another study conducted among 13–17 year old urban Portuguese adolescents showed that increased breakfast frequency had beneficial effects on a reducing overweight and obesity [56].

These findings are different from those observed among racially diverse, urban adolescents (Minneapolis-USA) enrolled in the Team COOL (Controlling overweight and obesity for life), with a mean age of 17.2 years, where participants who reported never eating family dinner were significantly more likely to be overweight [26]. Eating meals with family or alone was found to have no significant association to obesity in boys and girls aged 12–17 years of age, in a Dubai based study [50].

Dietary habits and eating practices within the community and school were not found to be significantly associated with the likelihood of being overweight and obese among early and mid-adolescents in the current study. This finding is similar to a study conducted among children aged 11–18 years, where there was no significant association between fast food consumption and BMI in females. However, the same study showed that males who consumed fast foods regularly (≥3 times per week) had significantly lower BMI compared with males who frequented fast food restaurants irregularly (≤2 times per week) [57]. Other cross-sectional and cohort studies showed significant association between fast food consumption, increased caloric intake [50,58], and higher BMI Z-scores [59]. Among girls aged 8–12 years of age, those who consumed fast foods ≥ 2 times a week at baseline, experienced the highest increase in mean BMI Z-scores compared to those who ate fast foods ≤ 1 times a week [60]. In another study where the effects of genetics vs. environment were disaggregated, twins living apart exhibited greater discordance in BMI changes, physical activity and fast food consumption from adolescence into adulthood, highlighting the influence of physical and household environments on BMI [61]. As these adolescents mature into adulthood, studies conducted among adults document a direct link between fast food consumption and increase in BMI [62,63,64].

It is interesting that irregular breakfast consumption on weekends and week days was generally higher among girls. However, the girls made significantly more tuck shop purchases than boys in the early adolescence group, and the frequency of tuck shop purchases was more regular among early- and mid-adolescent girls, who tend to gain weight. It seems that girls who skip breakfast might consume more food at other times, such as from tuck shop purchases and thereby gain more weight. Unhealthy snacking is an important feature of adolescent food consumption, and since many snacks have a high caloric content, the direct relationship between snacking and the risk of overweight and obesity observed in this study could indicate increased energy intake [65].

## 5. Conclusions

The current study findings identified similarities and differences in dietary habits and eating practices across gender, in early and mid-adolescence, from rural and urban sites, and in specific environments (home, community and school environment). Being male and residing within a rural setting in early and mid-adolescence was associated with reduced risk of being overweight and obesity. The irregular consumption of breakfast on weekdays was associated with the increased risk of being overweight and obesity among mid-adolescents, whereas irregular consumption of breakfast on weekend days was only associated with increased risk of overweight and obesity among mid-adolescent individuals. This data can inform targeted community based interventions for adolescent obesity prevention programmes, through understanding dietary habits in both rural and urban settings of South Africa. Future studies need to further investigate dietary practices within the home environment in order to develop interventions aimed at reducing the risk of overweight and obesity, especially among urban adolescents.

## Figures and Tables

**Table 1:** Anthropometric characteristics of Y13/EA and Y15/MA boys’ vs. girls in rural and urban participants (means and CIs where appropriate).

Site	Rural (Agincourt)		*p*-Value (Boys’ vs. Girls within Same Centre)	Urban (Soweto)		*p*-Value (Boys’ vs. Girls within Same Centre Across Centres)	*p*-Value (Boys’ vs. Boys Across Centres)	*p*-Value (Girls’ vs. Girls Across Centres)
Age Group	Y13/EA			Y13/EA				
Gender	Boys (*n* = 105)	Girls (*n* = 98)		Boys (*n* = 760)	Girls (*n* = 805)			
BMI (kg/m^2^) (mean 95 CI)	16.82	18.22	<0.001	18.64	20.64	<0.001	<0.001	<0.001
	(16.41–17.22)	(17.48–18.95)		(18.38–18.90)	(20.31–20.97)			
Underweight (%)	4.76	8.16	0.14 *	5.39	3.73	<0.001 *	<0.001	<0.001
Normal (%)	85.71	74.49		66.71	60.12			
Overweight (%)	3.81	11.22		6.05	15.03			
Obese (%)	5.71	6.12		21.84	21.12			
Height (cm) (mean 95 CI)	144.94	148.83	0.00	154.59	155.6	0.13	<0.001	<0.001
	(143.40–146.51)	(147.24–150.41)		(153.94–155.24)	(155.13–156.06)			
Weight (kg) (mean 95 CI)	35.62	40.72	0.00	44.87	50.16	<0.001	<0.001	<0.001
	(34.23–37.01)	(38.60–42.84)		(44.05–45.48)	(49.28–51.04)			
Age Group	Y15/MA			Y15/MA				
Gender	Boys (*n* = 89)	Girls (*n* = 100)		Boys (*n* = 747)	Girls (*n* = 786)			
BMI (kg/m^2^) (mean 95 CI)	18.9	21.43	0	19.62	22.27	<0.001	0.04	0.08
	(18.39–19.41)	(20.63–22.23)		(19.39–19.85)	(21.95–22.59)			
Underweight (%)	4.4	3.88	0.00 **	7.44	2.11	<0.001 *	0.11	0.20
Normal (%)	90.11	73.79		79.74	69.39			
Overweight (%)	1.1	16.5		5.51	16.98			
Obese (%)	4.4	5.83		7.31	11.52			
Height (cm) (mean 95 CI)	162.55	159.87	0.01	165.91	158.66	<0.001	<0.001	0.06
	(160.73–164.38)	(158.79–160.95)		(165.35–166.48)	(158.23–159.09)			
Weight (kg) (mean 95 CI)	50.38	54.82	0.00	54.21	56.09	<0.001	<0.001	0.32

WHO BMI-for-age Z-score classifications: Underweight = Z ≤ −2SD; Normal = Z > −2 ≤ +1SD; Overweight = Z > +1 ≤ +2SD Obese = Z > +2SD. Y13/EA = year 13/early-adolescents; Y15/MA = year 15/mid-adolescents; BMI = body mass index (kg/m^2^), Kg = kilogram; cm = centimetres; *p*-value based on Pearson Chi-squared test for categorical and t independent test for continuous variables * *p*-value based on Pearson Chi-squared test; ** *p*-value based on both Pearson Chi-squared and Fisher exact test. Significant differences set at *p* < 0.05.

**Table 2:** Comparison of dietary practices and eating habits between genders within same site in the Y13/EA group.

Environment	Indicators	Frequencies	Urban (Soweto) (%)			Rural (Agincourt) (%)		
			Males *n* = 760	Females *n* = 805	*p*-Value	Males *n* = 105	Females *n* = 98	*p*-Value
Home	Frequency of eating main with family in the previous week	Never	22.4	22.84	0.74	20.95	6.12	<0.001
		Some days	13.43	14.68		45.71	42.86	
		Almost everyday	64.1	62.48		33.33	51.02	
	Frequency of eating breakfast on weekend days in the previous week	Irregular (1 weekend day)	12.19	14.27	0.23	6.67	3.06	0.20
		Regular (both weekend days)	87.81	85.73		93.33	96.94	
	Frequency of eating breakfast on week days in the previous week	Irregular (≤2 days/week)	21.19	29.25	0.00	11.43	6.12	0.10
		Regular (3–5 days/week)	78.81	70.75		88.57	93.88	
	Frequency of consuming snacks whilst watching TV in the previous week	Irregular (≤3 times/week)	51.71	49.44	0.30	84.76	77.55	0.10
		Regular (>3 times/week)	48.29	50.56		15.24	22.45	
	Number of snacks consumed whilst watching TV during the previous week (mean ± SD)		4 ± 5.1	5 ± 5.6	0.02	1 ± 2.8	2 ± 3.8	0.12
Community	Frequency of fast foods items consumption during the previous week	Irregular (≤3 times/week)	1.97	1.47	0.70	5.71	10.2	0.20
		Regular (>3 times/week)	98.03	98.26		94.26	89.8	
	Number of fast food items consumed during the previous week (mean ± SD)		17 ± 8.6	17 ± 8.5	0.12	8 ± 4.8	9 ± 5.7	0.24
School	Frequency of tuck shop purchases during the previous week	Irregular (≤3 times/week)	14.08	8.94	<0.001	15.24	19.39	0.40
		Regular (>3 times/week)	85.92	91.06		84.76	80.61	
	Number of tuck shop items purchased in the previous week (mean ± SD)		12 ± 8.0	13 ± 7.9	<0.001	6 ± 4.3	7 ± 4.9	0.30

*p*-value based on Pearson Chi-squared test for categorical and *t*-independent test for continuous variables. Significant differences set at *p* < 0.05.

**Table 3:** Comparison of dietary practices and eating habits between genders within the same site in the Y15/MA group.

Environment	Indicators	Frequencies	Urban (Soweto) (%)			Rural (Agincourt) (%)		
			Males *n* = 747	Females *n* = 786	*p*-Value	Males *n* = 89	Females *n* = 100	*p*-Value
Home	Frequency of eating main with family in the previous week	Never	8.82	10.39	0.41	26.37	18.81	<0.001
		Some days	26.2	27.53		45.05	32.67	
		Almost everyday	64.98	62.08		28.57	48.51	
	Frequency of eating breakfast on weekend days in the previous week	Irregular (1 weekend day)	17.2	20.97	0.57	5.49	6.8	0.71
		Regular (both weekend days)	82.8	79.03		94.51	93.2	
	Frequency of eating breakfast on week days in the previous week	Irregular (≤2 days/week)	27.47	44.89	<0.001	10	13.59	0.44
		Regular (3–5 days/week)	72.53	55.11		90	86.41	
	Frequency of consuming snacks whilst watching TV in the previous week	Irregular (≤3 times/week)	1.03	2.11	0.08	1.1	0.97	0.93
		Regular (>3 times/week)	98.97	97.89		98.9	99.03	
	Number of snacks consumed whilst watching TV during the previous week (mean ± SD)		8 ± 5.9	9 ± 7.3	<0.001	5 ± 3.3	5 ± 2.9	0.98
Community	Frequency of fast foods items consumption during the previous week	Irregular (≤3 times/week)	3.21	2.35	0.30	9.89	9.7	0.96
		Regular (>3 times/week)	96.79	97.65		90.1	90.29	
	Number of fast food items consumed during the previous week (mean ± SD)		17 ± 8.9	18 ± 9.9	<0.001	7 ± 4.6	8 ± 6.0	0.20
School	Frequency of tuck shop purchases during the previous week	Irregular (≤3 times/week)	12.69	11.28	0.39	23.08	14.56	0.13
		Regular (>3 times/week)	87.31	88.72		76.92	85.44	
	Number of tuck shop items purchased in the previous week (mean ± SD)		12 ± 8.6	13 ± 9.5	0.07	9 ± 6.6	9 ± 6.5	0.52
	Frequency of lunch box usage during the previous week	Irregular (≤3 times/week)	92.25	81.9	<0.001	83.15	75	0.17
		Regular (>3 times/week)	7.75	18.1		16.8	25	

*p*-value based on Pearson chi squared test for categorical and t-independent test for continuous variables. Significant differences set at *p* < 0.05.

**Table 4:** Comparisons of dietary practices and eating habits between sites for Y13/EA and Y15/MA age groups.

Environment	Indicators	Frequencies	Y13/EA (% and Mean 95%CI)			Y15/MA (% and Mean 95% CI)		
			Urban (Soweto) *n* = 1565	Rural (Agincourt) *n* = 203	*p*-Value	Urban (Soweto) *n* = 1533	Rural (Agincourt) *n* = 189	*p*-Value
Home	Frequency of eating main with family in the previous week	Never	22.66	13.79	<0.001	9.62	22.4	<0.001
		Some days	14.07	44.33		26.88	38.54	
		Almost everyday	63.27	41.87		63.5	39.06	
	Frequency of breakfast consumption on weekend days in the previous week	Irregular (1 weekend day)	13.26	4.93	<0.001	19.11	6.19	<0.001
		Regular (both weekend days)	96.74	95.07		80.89	93.81	
	Frequency of breakfast consumption on week day in the previous week	Irregular (≤2 days/week)	25.34	8.87	<0.001	36.31	11.92	<0.001
		Regular (3–5 days/week)	74.66	91.13		63.69	88.08	
	Frequency of consuming snacks whilst watching TV in the previous week	Irregular (≤3 times/week)	50.54	81.28	<0.001	1.58	1.03	0.56
		Regular (>3 times/week)	49.46	18.72		98.42	98.97	
	Number of snacks consumed whilst watching during the previous week (mean ± SD)		4 ± 5.4	1 ± 3.3	<0.001	9 ± 6.7	5 ± 3.0	<0.001
Community	Frequency of fast foods items consumption during the previous week	Irregular (≤3 times/week)	1.85	7.88	<0.001	2.77	9.79	<0.001
		Regular (>3 times/week)	98.15	92.12		97.23	90.21	
	Number of fast foods items consumed during the previous week (mean ± SD)		17 ± 8.6	8 ± 5.2	<0.001	17 ± 9.4	8 ± 5.4	<0.001
School	Frequency of tuck shop purchases during the previous week	Irregular (≤3 times/week)	11.44	17.24	0.02	13.55	18.56	0.06
		Regular (>3 times/week)	88.86	82.76		86.45	81.44	
	Number of tuck shop items purchased in the previous week (mean ± SD)		12 ± 7.9	7 ± 4.6	<0.001	12 ± 9.0	9 ± 6.5	<0.001
	Frequency of lunch box usage during the previous week	Irregular (≤3 times/week)	82.1	61.19	<0.001	86.98	78.84	<0.001
		Regular (>3 times/week)	17.9	38.81		13.02	21.16	

*p*-value based on Pearson Chi-squared test for categorical and t-independent test for continuous variables; Significant differences set at *p* < 0.05.

**Table 5:** Multiple logistic regression analysis for risk of being overweight or obese (BMI for age Z-scores cut offs), gender, site and dietary practices.

BMI Z-Scores Y13/EA			M1		M2			M3		M4
			Exp (B) (95% CI)	*p*-Value	Exp (B) (95% CI)	*p*-Value	Exp (B) (95% CI)	*p*-Value	Exp (B) (95% CI)	*p*-Value
Sex			0.402 (0.301–0.538)	<0.001	0.399 (0.298–0.535)	<0.001	0.402 (0.300–0.538)	<0.001	0.401 (0.299–0.537)	<0.001
Female			1		1		1		1	
Site			0.604 (0.367–0.995)	0.60	0.558 (0.332–0.939)	0.03	0.580 (0.349–0.964)	0.04	0.547 (0.324–0.924)	0.02
Urban			1		1		1		1	
Dietary Practices/Habits										
Home	Frequency of eating main meal...	Never	1		1		1		1	
		Some days	0.555 (0.349–0.885)	0.01	1.1779 (1.115–2.836)	0.02	1.794 (1.126–2.860)	0.01	1.777 (1.114–2.835)	0.02
		Almost everyday	0.611 (0.421–0.888)	0.01	1.627 (1.119–2.366)	0.01	1.624 (1.117–2.361)	0.01	1.610 (1.106–2.343)	0.013
	Frequency of eating breakfast on weekend...	Regular	1		1		1		1	
		Irregular	1.210 (0.813–1.803)	0.35	1.213 (0.814–1.807)	0.34	1.210 (0.812–1.803)	0.35	1.216 (0.815–1.813)	0.34
	Frequency of eating breakfast on week days...	Regular	1		1		1		1	
		Irregular	1.399 (1.021–1.917)	0.04	1.385 (1.010–1.900)	0.04	1.390 (1.014–1.906)	0.04	1.382 (1.007–1.896)	0.05
	Frequency of consuming snacks whilst watching TV...	Regular	1		1		1		1	
		Irregular	1.289 (0.975–1.704)	0.07	0.741 (0.494–1.110)	0.15	0.761 (0.506–1.145)	0.19	0.761 (0.506–1.145)	0.19
	Number of snacks consumed whilst watching TV...				0.998 (0.961–1.036)	0.90	0.999 (0.961–1.038)	0.97	1.001 (0.963–1.041)	0.96
Community	Frequency of fast foods items consumption...	Regular			1				1	
		Irregular			1.327 (0.502–3.508)	0.57			1.327 (0.501–3.518)	0.56
	Number of fast food items consumed...				0.993 (0.976–1.011)	0.44			0.994 (0.976–1.012)	0.52
School	Frequency of tuck shop purchases...	Regular					1		1	
		Irregular					0.739 (0.423–1.288)	0.29	0.742 (0.425–1.294)	0.29
	Number of tuck shop items purchased...						0.989 (0.967–1.011)	0.33	0.991 (0.969–1.014)	0.45
R2 values of each model			0.074		0.076		0.076		0.077	

We performed multiple logistic regression analysis for being overweight and obese (cut offs set by using BMI for age Z-scores) with gender, centre and dietary practices and eating habits in different settings (home, community and environment) at Y13/EA. Significant differences set at *p* < 0.05. Model 1 adjusted for the crude model and dietary habits and eating practices within the home. Model 2 was adjusted for Model 1 and dietary habits and eating practices within the community. Model 3 was adjusted for Model 2 and dietary habits and eating practices within the school. Model 4 was adjusted for Model 3 and dietary habits and eating practices within the school.

**Table 6:** Multiple logistic regression analysis for risk of being overweight or obese (BMI for age Z-score cut-offs), gender, site and dietary practices in different settings among Y15/MA adolescents.

BMI Z-scores Y15/MA			M1		M2		M3		M4	
			Exp (B) (95% CI)	*p*-Value	Exp (B) (95% CI)	*p*-Value	Exp (B) (95% CI)	*p*-Value	Exp (B) (95% CI)	*p*-Value
Sex			0.285 (0.212–0.383)	<0.001	0.283 (0.211–0.381)	<0.001	0.296 (0.219–0.399)	<0.001	0.294 (0.218–0.397)	<0.001
Female			1		1		1		1	
Site			0.688 (0.424–1.118)	0.13	0.650 (0.394–1.073)	0.09	0.703 (0.431–1.147)	0.16	0.666 (0.403–1.102)	0.11
Urban			1		1		1		1	
Dietary practices/habits										
Home	Frequency of eating main meal...	Never	1		1		1		1	
		Some days	0.876 (0.554–1.387)	0.57	0.873 (0.551–1.382)	0.56	0.882 (0.551–1.411)	0.60	0.875 (0.546–1.401)	0.58
		Almost everyday	1.091 (0.719–1.656)	0.68	1.081 (0.712–1.641)	0.72	1.097 (0.715–1.682)	0.67	1.082 (0.705–1.660)	0.72
	Frequency of eating breakfast on weekend...	Regular	1		1		1		1	
		Irregular	1.476 (1.065–2.047)	0.02	1.467 (1.058–2.035)	0.02	1.538 (1.105–2.141)	0.01	1.530 (1.099–2.129)	0.01
	Frequency of eating breakfast on week days…	Regular	1		1		1		1	
		Irregular	1.148 (0.867–1.521)	0.34	1.140 (0.860–1.511)	0.36	1.121 (0.841–1.495)	0.44	1.115 (0.836–1.487)	0.46
	Frequency of consuming snacks whilst watching TV…	Regular	1		1		1		1	
		Irregular	0.763 (0.214–2.721)	0.68	0.773 (0.217–2.757)	0.69	0.792 (0.221–2.841)	0.72	0.802 (0.223–2.877)	0.73
	Number of snacks consumed whilst watching…		1.00 (0.980–1.020)	0.97	1.005 (0.983–1.028)	0.65	0.996 (0.974–1.018)	0.71	1.000 (0.977–1.024)	1.00
Community	Frequency of fast foods items consumption...	Regular			1				1	
		Irregular			0.838 (0.369–1.902)	0.67			0.779 (0.327–1.855)	0.57
	Number of fast food items consumed…				0.991 (0.975–1.008)	0.30			0.990 (0.972–1.008)	0.28
School	Frequency of tuck shop purchases...	Regular					1		1	
		Irregular					0.738 (0.444–1.227)	0.24	0.762 (1.457–1.272)	0.30
	Number of tuck shop items purchased...						1.002 (0.984–1.021)	0.79	1.006 (0.987–1.026)	0.54
	Frequency of bringing lunch box...	Regular					1		1	
		Irregular					0.815 (0.562–1.183)	0.28	0.821 (0.566–1.192)	0.30
R2 values of each model			0.098		0.099		0.098		0.1	

We performed multiple logistic regression analysis for being overweight and obese (cut offs set by using BMI for age Z-scores) with gender, centre, and dietary practices and eating habits in different settings (home, community and environment) at Y15/MA. Significant differences were set at *p* < 0.05. Model 1 was adjusted for the crude model and dietary habits and eating practices within the home. Model 2 was adjusted for Model 1 and dietary habits and eating practices within the community. Model 3 was adjusted for Model 2 and dietary habits and eating practices within the school. Model 4 was adjusted for Model 3 and dietary habits and eating practices within the school.
